# Children’s Temperament: A Bridge between Mothers’ Parenting and Aggression

**DOI:** 10.3390/ijerph17176382

**Published:** 2020-09-02

**Authors:** Miguel A. Carrasco, Begoña Delgado, Francisco Pablo Holgado-Tello

**Affiliations:** Faculty of Psychology, Universidad Nacional de Educación a Distancia (UNED), Campus Norte, Calle de Juan del Rosal, 10, 28040 Madrid, Spain; macarrasco@psi.uned.es (M.A.C.); bdelgado@psi.uned.es (B.D.)

**Keywords:** aggression, discipline, communication, temperament, childhood, gender

## Abstract

Childhood aggression is important to acknowledge due to its social impact and importance in predicting future problems. The temperament of a child and parental socialization have been essential in explaining behavioral problems, particularly in the case of childhood aggression. The aim of this study is to examine—from the parents’ perspective—the role of childhood temperament in the dynamic by which mothers’ reactions socialize their children’s aggression. We also explore how children’s gender and age differences affect these relationships. The sample was composed of 904 participants between 1 and 6 years old. The Early Childhood Behavior Questionnaire and the Children’s Behavior Questionnaire were used to evaluate children’s negative affect and effortful control. The Parent–Child Relationship Inventory Maternal was used to assess maternal communication and discipline, and child aggression was assessed using the Children’s Behavior Checklist. The results supported the mediating role of temperament in the processes by which perceived mothers’ reactions socialize their children’s aggression and suggested that maternal behaviors may not have the same consequences for girls and boys. Specifically, the aggressiveness of girls is dependent on a negative affect throughout toddlerhood and early childhood, while for boys, the duration of the negative affect’s contribution is shorter, and aggressiveness is more sensitive to the maternal behaviors of discipline and communication.

## 1. Introduction

Childhood aggression and its social impact play important roles in predicting future externalizing problems when children become adolescents or adults [1,2]. Studies of physical aggression show that by 17 months of age, the large majority of children are physically aggressive toward others (i.e., siblings, peers, and adults), and during preschool years, children start learning to regulate their use of aggression through different alternatives [3,4].

Child temperament and parental socialization have been established as potential mechanisms that contribute to behavioral problems [5,6]. Both temperament and parenting not only seem to contribute uniquely and simultaneously to children’s behavioral and emotional adjustment, but they can also both affect each other in a bidirectional process that may contribute to exacerbating or inhibiting behavioral problems [7,8,9,10]. Parents can shape children’s temperament and emotional and self-regulatory characteristics, which in turn are key predictors of children’s adjustment [11,12,13]. In turn, a child’s difficult temperament traits and behavioral problems may affect a parent’s educative practices and wellbeing [14].

Parenting behavior has historically been conceptualized as having two dimensions: parental control behaviors, including discipline, monitoring and autonomy granting; and parental positive affect toward the child, including warmth, acceptance, empathy, involvement and responsiveness [9,15,16]. Both have consistently emerged as predictors of children’s adjustment. The affective quality of the parent–child relationship has been related to lower levels of emotional and behavioral problems, particularly aggression [17,18,19,20]. This dimension also tends to facilitate children’s positive emotions, discourage negative affects and develop effective emotional regulation in infants [21,22]. Likewise, positive parental control has also been related to children’s adjustment. Parents characterized by firm decisions and warm family relationships have children who show higher levels of social adjustment [23].

Nevertheless, parental behaviors have complex relationships with children’s adjustments, and their effects on child behavior seem to depend on the positive or negative quality of parenting practices and the children’s characteristics. Moreover, these dynamics may be highly individualized. For example, Degnan, Calkins, Keane, and Hill-Soderlund [24] found that maternal control was associated with an increase in behavioral problems in highly reactive children, whereas for children with high regulation, maternal control was beneficial. The degree to which parental control behaviors shape children’s difficult temperament or negative emotionality presents a complex question, which has been the focus of different studies and which prevents us from drawing simple or linear relationships [25].

Previous research has also revealed children’s negative emotionality and effortful control as two important dimensions of temperament that predict the externalization and internalization of psychological problems [26,27,28,29] and that specifically predict aggression [30,31,32]. Children who are unable to modulate their negative affects are likely to become physiologically over-aroused and to behave in ways that may undermine the quality of social interactions [14,33,34,35,36].

In transactional models, in which the goal is to clarify the degree to which parenting shapes temperament and vice versa, evidence suggests that this dynamic may operate in both directions (for a review, see Kiff et al. [9]); that is, even when temperament is considered to be genetically based and relatively stable, evidence shows that it can be shaped by context, experience and social interactions [37]. Specifically, parenting communication and discipline behaviors can help children to maintain an optimal level of emotional arousal and teach them to regulate their internal states; conversely, they can contribute to children’s emotional over-arousal and dysregulation [38,39]. Consequently, the increase or decrease in children’s over-arousal and regulation can exacerbate or inhibit aggression [40,41]. Therefore, temperament may be a process through which maternal attitudes and reactions can affect childhood aggression.

There is convincingly substantial evidence for the role of child temperament as a moderator of the relationship between parenting and children’s problems (i.e., children higher in negative affects or with a difficult temperament demonstrate an increased risk of adjustment problems in the presence of poor parenting). However, in spite of the fact that studies of bidirectional and interactive effects highlight both mediational and conditional processes, less research has been conducted examining the role of temperament as a mediator between parenting and children’s problems. Such research might account for the complexity of the developmental processes leading to maladjustment. Thus, our primary focus in this study is the exploration of how mothers’ reactions to children’s temperament characteristics (i.e., negative affect and effortful control) may explain aggression in infancy and early childhood. Specifically, the aims of this study are to analyze the direct effects of perceived maternal communication and discipline on children’s negative emotionality, effortful control and aggressive behavior and to analyze the mediating effects of children’s temperament on mothers’ parenting and children’s aggressive behavior from the parents’ perspective.

Finally, the meaning and impact of different childhood temperaments and parenting behaviors might be dependent on the children’s developmental stage and gender. Regarding the child’s age as a moderator, research has shown that parental responsiveness (including warmth) consistently predicts developmental increases in effortful control during childhood [9,42,43,44]. Therefore, it could be expected that mediating relationships may be more likely later in childhood as temperament becomes more stable [45]. Previous research also leads us to consider gender as a significant moderator of childhood aggression. Parenting practices sometimes vary by gender [46], and child aggression tends to be higher in boys than in girls and tends to increase between the ages of 1 and 6 years [4,47]. In addition, girls tend to exhibit more effortful control than boys [48,49], so we could expect that the mediating effects of temperament might operate differently for boys and girls. Since previous research leads us to consider sex and age as significant moderators of childhood aggression, we also explore the moderating effects of children’s sex and age on the aforementioned direct and mediating relationships.

In summary, the aim of this study is to explore the extent to which parenting factors can contribute to the control of childhood aggression both directly and through the potential effect of the child’s temperamental variables. Specifically, it is expected that maternal discipline and communication mitigate childhood aggressiveness and that mothers’ parenting shapes children’s temperament (reducing their negative affect and improving their effortful control), and that this mediated effect should become clearer in older children.

## 2. Materials and Methods

### 2.1. Participants

The total number of participants was 904. The sample was divided into two groups based on the following developmental criteria: 482 toddlers aged 1 to 3 years old (52.3% boys) with an average age of 26.70 months and a standard deviation of 7.29, and 422 young children between 3 and 6 years of age (42.42% boys) with a mean age of 51.32 months and a standard deviation of 11.29. The sample was selected using probabilistic cluster sampling, with the school serving as the sampling unit. Using this procedure, 11 public and semi-private early education schools were randomly selected from all such schools in the Community of Madrid. The entire sample was composed of Spanish children. This research was authorized by the Department of Education and the directors of the schools in which it was conducted. Families’ participation in the study was voluntary, and they were not compensated or otherwise rewarded for their collaboration. Data for this study were collected between 2010 and 2011

The families’ socioeconomic status, estimated using the Hollinshead Index [50], showed that 72.4% of the fathers and 73.2% of the mothers were middle-class. The majority of families were intact, comprising both a mother and father (85%).

### 2.2. Instruments

Two forms were used to evaluate temperament—one for each age group studied. The two were conceptually similar and were derived from the temperament model developed by Rothbart and her collaborators [51,52,53]. The content of the items referred to the child’s behavior in numerous everyday situations and at play, as well as in childcare settings. Items were evaluated with a Likert-type scale, with seven response options assessing the frequency (from 1 = “never” to 7 = “all the time”) of different occurrences during the week prior to the date on which the form was filled out. Mothers were asked to respond based on the occurrence of behaviors during the week preceding the date on which the questionnaire was filled out.

The Early Childhood Behavior Questionnaire (ECBQ) [52,53,54] is designed for participants from 1 to 3 years of age. It consists of 201 items grouped into six dimensions that are obtained through exploratory factor analysis using the principal components extraction method and varimax rotation. The first dimension—perceptual sensitivity—measures the temperament characteristics relating to the ability to detect weak, low-intensity stimuli in the external environment, the child’s approach tendency, their quick orientation and the tendency to sustain attention to objects and events in the environment (43 items; Cronbach’s alpha = 0.88); frustration/negative affect assesses the tendency for the child to experience negative emotions towards limitations, sadness, high arousal and low inhibition (39 items; Cronbach’s alpha = 0.90); cuddliness evaluates the child’s ability to enjoy affection and caresses and the tendency to mold their body to the caretaker holding or embracing them (17 items; Cronbach’s alpha = 0.78); withdrawal/shyness addresses the child’s tendency to experience fear, shyness, and ill-being (23 items; Cronbach’s alpha = 0.85); motor activity measures the child’s motor expression and body movements (20 items; Cronbach’s alpha = 0.76); and impulsivity measures the child’s tendency to respond quickly by emitting responses or initiating a behavior (19 items; Cronbach’s alpha = 0.76). There has been ample evidence for the scale’s validity in a Spanish population [54]. According to the temperament model proposed by Rothbart [55], perceptual sensitivity and cuddliness are included as regulatory processes within the effortful control broad scale. Measures of reactivity, within which we distinguish measures of negative emotionality and withdrawal/shyness, are included in the negative affect broad scale.

The Children’s Behavior Questionnaire (CBQ) [53,54] is designed for participants aged 3 to 8 years old. It consists of 195 items with six dimensions extracted from exploratory factor analysis using the principal components extraction method and varimax rotation. Effortful control measures the ability to plan and suppress approach responses when instructed or in situations of novelty or uncertainty (39 items; Cronbach’s alpha = 0.91); low reactivity indicates a lack of expressiveness or the manifestation of emotions of a positive affect and a lack of sensitivity or ability to enjoy or derive pleasure from low-intensity stimuli (20 items; Cronbach’s alpha = 0.80); negative affect/frustration measures the child’s tendency to express anger and ill-being when confronted with limitations—this also includes the child’s tendency to experience negative emotions of fear and sadness (41 items; Cronbach’s alpha = 0.89); shyness refers to a child’s low or inhibited approach tendency in situations of novelty or uncertainty (18 items; Cronbach’s alpha = 0.92); high-intensity pleasure is defined as the amount of pleasure or enjoyment derived from high-intensity stimuli or activities that imply a high risk at that age (15 items; Cronbach’s alpha = 0.81); and perceptual sensitivity/smiling includes temperament characteristics related to being able to detect weak, low-intensity stimuli in the external environment—this includes a positive affect, pleasure, and enjoyment in response to change (22 items; Cronbach’s alpha = 0.81). As in the case with the ECBQ, there is adequate evidence for this instrument’s validity in a Spanish population [54].

In the temperament model proposed by Rothbart [55], attentional control, effortful control and cuddliness scales are included as regulatory processes within the broad effortful control scale. Measures of reactivity, within which we distinguish measures of negative emotionality (frustration, fear, ill-being, sadness and withdrawal/shyness), are included in the broad negative affect scale. Lastly, the extraversion–surgency broad scale is an aggregate of the measures of motor activity, impulsivity, perceptual sensitivity and high-intensity pleasure. For the purpose of this study, we used the two first broad scales: effortful control and negative affect.

The following two questionnaires were utilized to assess child aggression and parenting, respectively.

The Children’s Behavior Checklist Parent Report Form (CBCL) [56] is an inventory for parents that is based on the Achenbach System of Empirically-Based Assessment (ASEBA). We focused on the inventory’s checklist, which assesses children’s behavioral and emotional problems. The CBCL contains 100 items measured on a Likert scale of three points: 0 = not true, 1 = somewhat or sometimes true and 2 = very true or often true. This instrument provides information on different psychological syndromes that have been empirically obtained by factorial analysis. Mothers filled out the full questionnaire; however, for the purposes of this study, we only considered the Aggressive Behavior Scale (19 items; e.g., “demanding”, “can’t wait”, “fights”). The scale’s internal consistency for the two age groups of 1–3 years old and 3–6 years old is 0.73 and 0.84, respectively.

The Parent–Child Relationship Inventory (PCRI-M) [57,58] consists of 78 items assessing a mother’s attitude towards parenting and her children. Each item is measured on a Likert scale (0 = “not applicable”, and from 1 = “strongly agree” to 4 = “strongly disagree”). The inventory comprises eight scales: support, satisfaction with parenting, involvement, communication, discipline/limit setting, autonomy, role orientation and social desirability. For the purpose of this study, we used two of these scales: the communication scale assesses how the mother perceives her communication with her son/daughter to be effective (nine items—e.g., “My child generally tells me when something is bothering him or her”—the scale’s internal consistency for the two age groups of 1–3 years old and 3–6 years old is 0.70 and 0.71, respectively); discipline/limit setting focuses on a parent’s experience in disciplining a child, referring to the ability of parents to create and maintain parameters around a child’s behavior (12 items—e.g., “I have trouble disciplining my child” (reverse scoring)—the scale’s internal consistency for each age group is 0.61 and 0.68, respectively).

### 2.3. Procedure

Prior to sample selection, we sought authorization from the Community of Madrid Department of Education and the school directors and boards. At the time of those meetings, we sought parents’ informed consent to participate in the study. We then collected information from the children and their families using questionnaires filled out from the mothers’ perspective for each child (one questionnaire per child). Once completed, the questionnaires were given to the researchers to be corrected and analyzed. After the study, all parents received a report with their child’s data.

### 2.4. Statistical Analysis

First, we carried out a preliminary analysis in which the correlations between the variables and the basic descriptive statistics of the variables were obtained. Second, the mediating effects were analyzed in three steps using path analysis [59,60]. For all analyses, we used the statistical program linear structural relations, LISREL 8.8 software (Scientific Software International, Inc.: Skokie, IL, USA) [61].

## 3. Results

### 3.1. Basic Descriptive Statistics and Correlational Analysis in the Total Sample and by Gender

The descriptive statistics for all study variables in the total sample (1–3 years old) are shown in Table 1. There were significant differences in negative affect and aggressive behavior according to gender; specifically, the girls scored lower in these variables.

We also calculated correlations among variables in the total sample (1–3 years old) and by gender (Table 2). In the whole sample, in accordance with our expectations, negative affect and aggression showed a strong positive association. We also found a high negative correlation between effortful control and aggression, and effortful control and negative affect. By gender, the same pattern was found; however, the negative relation between effortful control and aggression was shown to be higher in girls.

In the sample of 3–6 years old the descriptive statistics for all analyses are shown in Table 3. There were significant differences in terms of aggressive behavior and effortful control according to gender; specifically, the girls scored lower in aggression and boys in effortful control.

Regarding the correlations among variables in the total sample (3–6 years old) and by gender (Table 4) for the whole sample, in accordance with our expectations, negative affect and aggression showed a strong positive association, as well as effortful control and discipline. We also found a high negative correlation between effortful control and aggression, as well as effortful control and negative affect. For gender, the same pattern was exhibited; however, the negative relation between effortful control and aggression was found to be higher in boys.

### 3.2. Mediational Analysis

**Figure 1:**
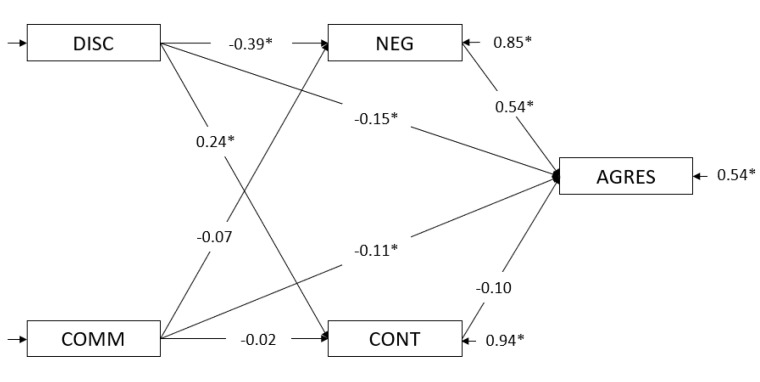
Mediational model for the 1–3-year-old group. Note: DISC = discipline; COMM = communication; NEG = negative affect; CONT = effortful control; AGRES = aggressive behavior. * *p* > 0.05.

In order to analyze the mediating effects of children’s temperament on maternal parenting and childhood aggression, we tested one model that included discipline and communication as antecedents, negative affect and effortful control as mediators and children’s aggressive behavior as the criterion variable (see Figure 1).

#### 3.2.1. Direct and Mediating Effects in the Total Sample of 1–3 Year-Olds

The statistical relationships between antecedents (discipline and communication) and aggressive behavior were statistically significant (Model 1.1) after constraining all indirect paths to zero. Specifically, significant direct effects were found for discipline (*c* = −0.39, Critical ratio (hereafter, *CR)* = −6.35) and communication (*c* = −0.15, *CR* = −2.42). The fit indices for Model 1.1 are presented in Table 5. Moreover, in step two of the model, we tested negative affect and effortful control (mediators) predicted by discipline and communication when the paths between the antecedents and the child’s problems were constrained to zero (Model 1.2): the values for negative affect from discipline were *a* = −0.39 and *CR* = −6.27, and those for effortful control from discipline were *a* = 0.24 and *CR* = 3.72. On the other hand, we also determined the negative affect from communication (*a* = −0.07, *CR* = −1.08) and effortful control from communication (*a* = −0.02, *CR* = −0.32). The paths were significant for discipline but not for communication, meaning that communication was not a relevant variable in the sample of participants between 1 and 3 years old. All these parameters were the same as those represented in Figure 1, which corresponds to step three of the model.

We also assessed a mediation model in which all parameters were allowed to vary (unconstrained model, Model 1.3; Figure 1). In this model, discipline and communication predicted aggressive behavior, mediated by the temperament variables (negative affect and effortful control). In Model 1.3, we found a decrease in the magnitude of the direct paths from the antecedents (discipline and communication) to aggressive behavior (Model 1.1). These results indicate a partial mediation: in Model 1.1, the direct path between discipline and aggressive behavior was −0.39, whereas in Model 1.3 it was −0.15, and the path from communication to aggressive behavior showed a small decrease of 0.04 points (from −0.15 to −0.11). Both paths in Model 1.3 remained statistically significant. Regarding the direct path from the mediators to aggressive behavior, the relationship between negative affect and aggressive behavior was significant (*a* = 0.54, *CR* = 7.23), but the path of effortful control was not (*a* = −0.10, *CR* = −1.67). The fit indices for Model 1.3 are presented in Table 5.

As indicated in Figure 1, this model represents a partial mediation of discipline on aggressive behavior, mainly through a negative affect. In this sense, the total indirect effect of discipline on aggressive behavior, which is basically composed of a negative affect (IF = −0.21), was significant (IF = −0.23; CR = −5.44). Thus, the relationship between discipline and aggressive behavior was partially mediated by negative affect but not by effortful control. As we can see in Table 5, the increase in the chi-square test suggests an improvement of the goodness of fit in the unrestricted model (Model 1.3) in comparison to the previous model. In addition to the aforementioned indicators, the expected cross-validation index (ECVI) and the Consistent Akaike Information Criterion (CAIC) were also calculated, and both indices were used to measure the comparative fit between two or more models, with smaller values being considered to represent the best fit [62]. In the mediational analysis, the global fit indices are secondary because the objective is to probe the mediational role of the variables of interest. In this sense, we can obtain adequate fit indices in the unrestricted models simply by correlating the error terms of the mediational variables.

##### Direct and Mediating Effects in the Sample of 1–3 Year-Olds by Gender

Among boys, the statistical relationships between discipline and communication and aggressive behavior were statistically significant (Model 1.1 boys) (discipline: *c* = −0.42, *CR* = −5.19; and communication: *c* = −0.21, *CR* = −2.61). The fit indices for boys in Model 1.1 are presented in Table 5. Among girls (Model 1.1 girls), discipline was significant in predicting aggressive behavior (*c* = −0.34, *CR* = −3.92), but communication was not (*c* = −0.08, *CR* = −0.93).

**Figure 2:**
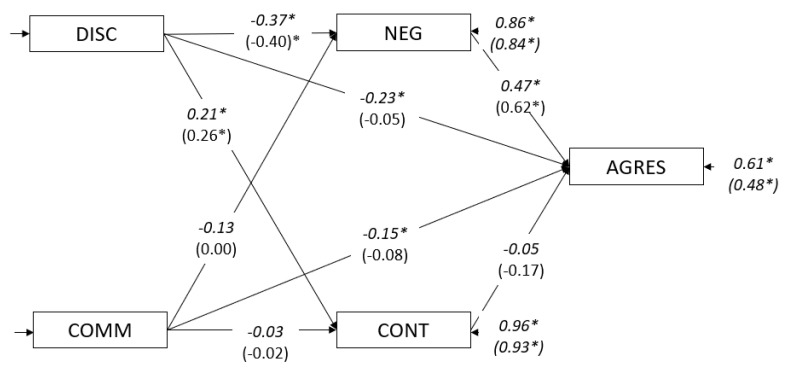
Mediational model for 1–3 year-olds by gender. Note: DISC = discipline; COMM = communication; NEG = negative affect; CONT = effortful control; AGRES = aggressive behavior. Values in italics are for boys; values in parentheses are for girls. * *p* > 0.05.

The parameter values and fit indices of the second step (Model 1.2 boys and Model 1.2 girls) are shown in Figure 2 and Table 5.

In step three (Figure 2), the results for boys (Model 1.3 boys) indicated a partial mediation: in Model 1.1 boys, the direct path between discipline and aggressive behavior was −0.42, while in Model 1.3 boys, it was −0.23. The path from communication to aggressive behavior showed a small decrease of 0.06 points (from −0.21 to −0.15). Both paths for Model 1.3 boys remained statistically significant. Regarding the direct path from the mediators to aggressive behavior, the relationship between negative affect and aggressive behavior was significant (*a* = 0.47, *CR* = 5.08), but the path of effortful control was not (*a* = −0.05, *CR* = −0.63). The fit indices for Model 1.3 are presented in Table 5.

For girls (Model 1.3 girls), the results indicated a total mediation for discipline through negative affect. In Model 1.1 girls, the direct path between discipline and aggressive behavior was −0.34, while in Model 1.3 girls, it was −0.05 and was no longer significant (*CR* = −0.56). Regarding the direct path from the mediators to aggressive behavior, the relationship between negative affect and aggressive behavior was significant (*a* = 0.62, *CR* = 4.37), but the path of effortful control was not (*a* = −0.17, *CR* = −1.49). The fit indices for Model 1.3 girls are presented in Table 5.

As indicated in Figure 2, this model mainly presented a partial mediation of discipline on aggressive behavior through a negative affect in boys, but a total mediation was found in girls. In this sense, the total indirect effect of discipline on aggressive behavior, which is basically composed of a negative affect (IF = 0.21), is significant (IF = −0.23; CR = −5.44). Thus, relationships between discipline and aggressive behavior were partially mediated by a negative affect but not by effortful control.

In boys and girls between 1 and 3 years old, discipline tends to decrease negative affect, subsequently decreasing aggressive behavior. However, some differences were found: (a) the direct path between discipline and aggressive behavior was still significant in boys but not in girls, and (b) the total effect of communication in reducing aggressive behavior was significant in boys, but not in girls. In other words, for girls, discipline is most effective through negative affect, and communication has no effect when negative affect is present in the model.

##### Direct and Mediating Effects in the Total Sample of 3–6-Year-Olds

**Figure 3:**
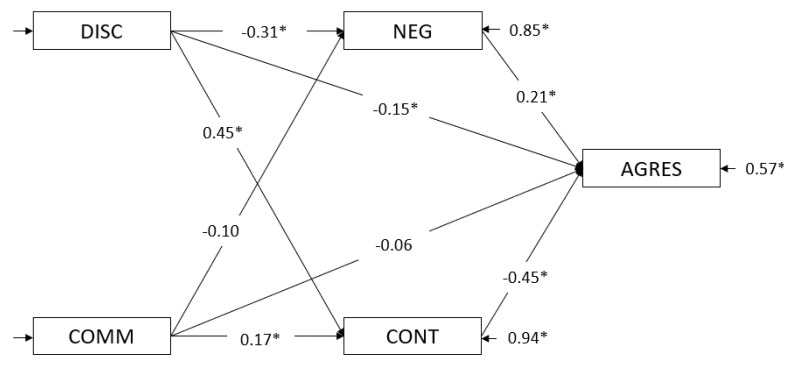
Mediational model for the 3–6-year-old group. Note: DISC = discipline; COMM = communication; NEG = negative affect; CONT = effortful control; AGRES = aggressive behavior. * *p* > 0.05.

The statistical relationships between discipline and communication and aggressive behavior were statistically significant (Model 2.1) after constraining all indirect paths (discipline: *c* = −0.42, *CR* = −7.74; communication: *c* = −0.15, *CR* = −2.85). The parameter values and fit indices of the second step (Model 2.2) are shown in Figure 3 and Table 5.

In step three (Figure 3), discipline and communication predicted aggressive behavior, mediated by the temperament variables (negative affect and effortful control). In Model 2.3, we found a decrease in the magnitude of the direct paths from the antecedents (discipline and communication) to aggressive behavior (Model 2.1). These results indicated a partial mediation; that is, while in Model 2.1 the direct path between discipline and aggressive behavior was −0.42, in Model 2.3, it was −0.15. For communication, we found a total mediation, whereby the path from communication to aggressive behavior was −0.15 in Model 2.1 and −0.06 (not significant) in Model 2.3. Regarding the direct path from the mediators to aggressive behavior, we found that the relationship between negative affect and aggressive behavior (*a* = 0.21, *CR* = 3.75) and the path of effortful control (*a* = −0.45, *CR* = −6.70) were both significant.

As indicated in Figure 3, this model presents a partial mediation of discipline on aggressive behavior and a total mediation of communication on aggressive behavior, but unlike in the model for the 1–3-year-olds, effortful control plays a relevant role. Thus, the relationship between discipline and aggressive behavior was partially mediated by negative affect and effortful control. Furthermore, the relationship between communication and aggressive behavior was totally mediated by effortful control, in general. Discipline tended to decrease negative affect and subsequently decrease aggressive behavior. It also tended to increase effortful control; this variable provoked a decrease in aggressive behavior. Communication tended to increase effortful control and subsequently decreased aggressive behavior. The fit indices of the models are presented in Table 5.

##### Direct and Mediating Effects in the Sample of 3–6-Year-Olds by Gender

**Figure 4:**
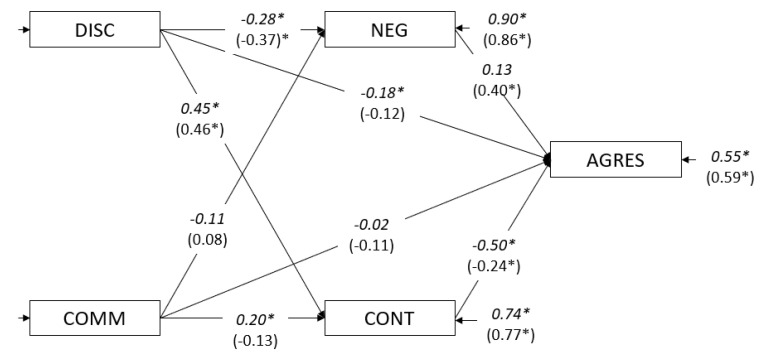
Mediational model for the 3–6-year-olds by gender. Note: DISC = discipline; COMM = communication; NEG = negative affect; CONT = effortful control; AGRES = aggressive behavior. Values in italics are for boys; values in parentheses are for girls. * p > 0.05.

In boys (Model 2.1 boys), the statistical relationships between discipline and communication and aggressive behavior were statistically significant only for the first variable (*c* = −0.44, *CR* = −5.00) and not for communication (*c* = −0.14, *CR* = −1.60). The fit indices for Model 2.1 boys are presented in Table 5. For girls (Model 2.1 girls), we found that discipline (*c* = −0.38, *CR* = −5.41) and communication (*c* = −0.17, *CR* = −2.50) were both significant in predicting aggressive behavior. The parameter values and fit indices for the second step (Model 2.2 boys and Model 2.2 girls) are shown in Figure 4 and Table 5.

In step three (Figure 4), for boys, the direct path between discipline and aggressive behavior was −0.44 in Model 2.1, while in Model 2.3, it was −0.18 (significant). The path between communication and aggressive behavior decreased from −0.14 to −0.02 and remained nonsignificant. Regarding the direct path from the mediators to aggressive behavior, the relationship between negative affect and aggressive behavior was not significant (*a* = 0.13, *CR* = 1.71), but it was significant for effortful control (*a* = −0.50, *CR* = −4.86). The fit indices for Model 2.3 are presented in Table 5.

Regarding girls, for Model 2.1 girls, the direct path between discipline and aggressive behavior was −0.38 (significant), while for Model 2.3 girls, it was −0.12 and was no longer significant (*CR* = −1.77). Communication predicted aggressive behavior (*c* = −0.17, *CR* = −2.50) for Model 2.1 girls; however, for Model 2.3 girls, this was not significant (*c* = −0.11, *CR* = −1.85). Regarding the direct path from the mediators to aggressive behavior, the relationship between negative affect and aggressive behavior was significant (*a* = 0.40, *CR* = 5.44), as was effortful control (*a* = −0.24, *CR* = −3.29). The fit indices for Model 2.3 girls are presented in Table 5.

As indicated in Figure 4, in boys, the model showed that discipline partially mediated aggressive behavior through a negative affect and effortful control, but in girls, discipline was totally mediated by negative affect, mainly, and by effortful control. On the other hand, communication was mediated by effortful control in boys but not in girls.

In boys and girls between 3 and 6 years old, discipline tended to decrease negative affect and subsequently decreased aggressive behavior (more evident in girls). Moreover, discipline increased effortful control, and then aggressive behavior decreased (more evident in boys). Communication tended to increase effortful control and subsequently decreased aggressive behavior, but only in boys. Some other relevant differences were found: (a) the direct path between discipline and aggressive behavior was still significant in boys but not in girls; (b) when temperament variables were included, communication became irrelevant in girls but not in boys, mainly through effortful control; and (c) in girls, the principal variable predicting aggressive behavior was a negative affect controlled by discipline, while in boys, it was effortful control improved by discipline and—to a lesser degree—by communication.

## 4. Discussion

This study simultaneously compared two parenting dimensions with two different temperament factors in relationship to childhood aggression during toddlerhood and early childhood. To date, few studies examining the effects of temperament and parenting on childhood aggression have compared both these two age groups and both genders. Thus, our results may help us to understand how children’s temperamental characteristics affect the way in which mother’s behaviors interact with their toddlers’ and young children’s aggression.

The first objective of this study was to explore the direct effects of perceived mothers’ communication and discipline on their children’s aggression and temperament (negative emotionality and effortful control). Our results are consistent with previous studies that have shown parental control and positive affect as two important predictors of children’s emotional and behavioral adjustment [9,15,16,23]. In our work, maternal discipline and communication were negatively and significantly related to children’s aggression, with one exception: maternal communication was not significantly related to aggression among toddler girls or with boys in young childhood. Therefore, in our study, maternal discipline emerged as a more efficient strategy than maternal communication to inhibit aggression.

It is interesting to note that maternal discipline is mainly focused on a child’s behavior, and aggression is also a behavioral and externalized manifestation. Accordingly, parental control strategies for 2 year-old children that include clear, consistent limits and non-punitive discipline lead those children to demonstrate better behavioral control and a capacity for delayed gratification when they are between 6 and 8 years old [40,63]. The relationship between maternal behavior and children’s temperament also underscores the effects of maternal behavior on children’s reactive and regulatory temperament traits, for which maternal discipline appears to be the most relevant factor. Maternal discipline (not communication) shows a significant direct effect on children’s negative affects and effortful control across group ages. In addition, maternal communication makes a significant contribution to effortful control (not negative affect) among older boys.

From a general view, our results are in line with previous research showing that parental behaviors can shape children’s temperament-related emotional and self-regulatory characteristics [24,63,64]. Specifically, they support previous findings concerning maternal discipline and communication and children’s positive emotions and effective emotional regulation [21,22]. Previous studies have also shown that toddlers appear to benefit from parental discipline practices, leading to increases in effortful control over a two-year period [65]. Parental responsiveness, in contrast, has consistently predicted developmental increases in effortful control in early childhood, but not earlier [44]. Our results show that a greater effect of maternal communication on older (3–6 years) children’s temperament may be in accordance with this developmental framework. The second aim of this study was to test the mediating effects of children’s temperament between mothers’ parenting and children’s aggression from the mothers’ perspective. Previous research has consistently confirmed that parental behaviors can shape children’s temperament and emotional and self-regulatory characteristics, which in turn affect children’s adjustment [10,13,24]. Specifically, maternal discipline and communication seem to facilitate children’s positive emotions, discourage negative affects and develop effective emotional regulation [21,22]. Our work confirms the mediating effects of temperament. Among toddlers, maternal discipline seems to shape a negative affect, and toddlers’ aggression then tends to decrease. Therefore, a temperamental toddler’s negative affect may be the potential process by which maternal discipline affects the toddler’s aggression. Furthermore, among young children (3–6 years old), a negative affect and effortful control were two significant mediators between maternal behavior and children’s aggression. Specifically, effortful control was a mediator between maternal communication and discipline and children’s aggression; furthermore, maternal communication was effective for mediating young children’s aggression only through its effect on effortful control.

Developmental qualities may explain the differences in the mediating effect across age groups that we observed in our study. Among toddlers, a negative affect is an essential component of aggression, especially anger and frustration [28,36,64,66]. However, as children grow from toddlerhood to around 3–4 years of age, they acquire effective emotional self-control, and at about 6 or 7 years old, the development of effortful control becomes more important [55,67]. Therefore, it can be said that, as childhood progresses, effortful control achieves a significant mediating effect. This result also highlights the importance of developing temperament dimensions to mediate the effects of mothers’ behaviors on children’s aggression.

The third aim of this study was to explore the moderating effects of children’s sex and age in the previously discussed direct and mediating relationships. Moderation effects were especially confirmed for maternal communication, since maternal communication was an effective strategy for reducing aggression in toddler boys and young girls. In contrast, perceived maternal discipline emerged as a robust strategy to deal with childhood aggression during the toddler and early childhood years. Mediating relationships were also affected by sex and age, suggesting that perceived maternal behaviors may operate through temperament differently according to gender and age. Specifically, maternal discipline became nonsignificant for toddlers’ and young girls’ aggression once temperament traits were considered. Therefore, maternal discipline was effective in reducing toddler girls’ aggression only through its inhibitory effect on a negative affect; likewise, perceived maternal discipline was effective for addressing older girls’ aggression only by reducing girls’ negative affects and by improving girls’ effortful control. In contrast, the mediational role of temperament traits was less intense for boys. After considering temperament traits, perceived maternal discipline remained significant for toddler boys’ aggression and for older boys’ aggression as reported by parents. Thus, aggression among boys may be more affected by maternal behaviors than girls’ aggression. Although this could be due to the smaller range of aggressive behaviors in the female sample, this result is in line with evidence showing a higher risk of externalized behaviors for boys exposed to lower levels of maternal sensitivity during early childhood [68]. Therefore, mediating relationships were confirmed not only in early childhood, but also in the toddler years. As previously mentioned, a negative affect has an important role in the toddler years, while effortful control acquires more relevance in early childhood. Unexpectedly, a negative affect remained important for young girls (but not young boys) as a mediator between maternal discipline and aggression. The direct and mediating relationships observed in this work prevent us from making simplistic interpretations; in this sense, it should be noted that, although girls showed less aggressiveness on average than boys and higher levels of effortful control on average than older boys, aggressiveness in older girls was still largely supported by a negative affect, and this aggressiveness was less sensitive to voluntary control or external control than it was in boys. Maternal discipline was the unique maternal behavior that affected girls’ aggression, mainly by reducing negative affects.

Our data suggest the need for a deeper understanding of the aspects surrounding the aggressive behavior of girls. Such behavior in toddlers and young girls, which is less frequent than in boys but has a greater dependence on a negative affect, may generate a stronger negative reaction in parents by violating their expectations about parenting to a greater extent. For example, Rolon-Arroyo, Arnold, Breaux and Harvey [69] found that, although challenging behaviors among preschoolers affect parental practices, only girls’ challenging behavior has the added effect of increasing parents’ negative affects. Similarly, Woodward et al. [70] found that, although girls tend to receive a more positive and warmer upbringing, this gender effect disappears when the child’s variables, such as their cognitive abilities or their affection, are taken into consideration. Reciprocally, it is well-known that the quality of childcare significantly affects child development, and this is especially true in the case of children with high levels of negative affect [11]. All of these factors should motivate researchers to pay greater attention to the aggressive behavior of girls and the effects of such behavior on parenting throughout the child’s development.

## 5. Conclusions

In summary, our study confirms that childhood aggression is affected by children’s temperament and parenting, but also that a child’s temperament factors mediate the way in which parenting is related to childhood aggression. Moreover, it seems that, in young children, parenting efforts became more relevant overall through their effects on children’s temperament (specifically, by mitigating negative affects and improving effortful control) than by their direct effects on childhood aggression. In addition, we found that the temperamental traits and aggressiveness of boys were more sensitive to the influence of maternal behaviors (especially maternal discipline) than the temperamental traits and aggressiveness of girls. Accordingly, we suggest that it is necessary to pay more attention to the specific features of girls’ aggression and to its effects on parental socialization practices.

We would like to note that the mediational model tested in this study is in agreement with the characteristics of the sample and the pragmatic objectives addressed. Obviously, depending on the specific objectives of the studies, other mediational models may be possible. For example, in the introductory section, we noted that extreme temperamental traits could even affect parenting modes. Consequently, the inverse mediational analysis could make sense in a sample of children with extreme temperament traits.

Finally, we would like to highlight that the previous conclusions need to be considered in light of certain limitations of our study, and that they will need to be confirmed by future research. First, a correlational and cross-sectional design was used; thus, the conclusions—especially the developmental ones—should be limited to this kind of design. In this regard, all potential causal relations between parenting and children temperament in this study must be exclusively considered from a statistical perspective, as a necessary but not sufficient condition [71]. Second, the present sample was composed primarily of middle-class, well-educated families of European descent who gave general consent to be contacted for participation in developmental research. Thus, the results obtained from more diverse populations may be different, and our findings must be generalized with caution. Third, all measures have been reported by mothers; in future, results must be considered from both parents’ perspectives. The significant relations between variables can be partially explained due to the shared variance of the method. Different results may be expected from other informants’ perspectives, and future studies must explore these results with a multi-informant approach. In this regard, it is important to highlight that fathers’ behaviors might be an important influence for children, and fathers’ perceptions of a child’s temperament and parenting may give different results that should be explored in future studies. Fourth, parents’ observations of their own child’s temperament and behavior must be assumed to be affected by their own parental characteristics (e.g., temperament, psychopathology, etc.), and the child’s behavior may also be highly affected by parenting and parental characteristics. As mentioned in the introduction, both temperament and parenting can also affect each other in a bidirectional process that may contribute to exacerbating or inhibiting behavioral problems [7,8,9,10]. Fifth, maternal reactions have been measured as a general attitude or parenting dimension instead of maternal reactions to the specific behaviors of specific children.

To end, we would like to state that future research could be addressed to explore this mediational model, and the moderating effect of children’s age and sex, in young children with highly difficult temperaments, oppositional disorder or others childhood factors that could be of interest [72,73]

## Figures and Tables

**Table 1:** Basic descriptive statistics in the total sample and by gender for the 1–3 year-old group.

	Total Sample		Boys		Girls	
	Mean	S.D.	Mean	S.D.	Mean	S.D.
DISC	30.31	4.66	30.03	4.65	30.62	4.66
COMM	22.74	2.70	22.74	2.78	22.73	2.62
NEG *	3.65	0.66	3.72	0.64	3.56	0.67
AGRES *	15.57	8.32	16.62	8.60	14.35	7.84
CONT	280.44	30.33	278.03	29.58	283.21	31.03

Note: DISC = discipline; COMM = communication; NEG = negative affect; CONT = effortful control; AGRES = aggressive behavior. * *p* < 0.05.

**Table 2:** Correlations in the total sample (above diagonal) and by gender (below diagonal) in the 1–3 year-old group.

	DISC	COMM	NEG	AGRES	CONT
DISC	---	−0.11 *	−0.37 **	−0.36 **	0.20 **
COMM	*0.09* (−0.11)	---	−0.05	−0.14 *	−0.04
NEG	−0.38 ** (−0.34 **)	*−0.09* (−0.01)	---	0.65 **	−0.43 **
AGRES	−0.39 ** (−0.30 **)	*−0.15* (−0.13)	0.61 ** (0.69 **)	---	−0.41 **
CONT	0.16 * (0.23 **)	*−0.04* (−0.03)	−0.39 ** (−0.47 **)	−0.31 ** (−0.53 **)	---

Note: DISC = discipline; COMM = communication; NEG = negative affect; CONT = effortful control; AGRES = aggressive behavior. Below the diagonal, the italicized values are for boys, and the values in parentheses are for girls. * *p* < 0.05.; ** *p* < 0.01.

**Table 3:** Basic descriptive statistics in the total sample and by gender for the 3–6 year-old group.

	Total Sample		Boys		Girls	
	Mean	S.D.	Mean	S.D.	Mean	S.D.
DISC	30.74	4.46	30.46	4.20	31.09	4.78
COMM	21.49	2.70	21.42	2.70	21.58	2.70
NEG	4.24	0.64	4.22	0.63	4.21	0.58
AGRES *	4.22	0.61	9.30	5.48	6.99	4.63
CONT *	8.38	5.26	4.12	0.65	4.41	0.58

Note: DISC = discipline; COMM = communication; NEG = negative affect; CONT = effortful control; AGRES = aggressive behavior. * Statistical difference between boys and girls (*p* < 0.05).

**Table 4:** Correlations in the total sample (above diagonal) and by gender (below diagonal) for the 3–6 year-old group.

	DISC	COMM	NEG	AGRES	CONT
DISC	---	0.08	−0.32 **	−0.43 **	0.46 **
COMM	*−0.11* (0.03)	---	−0.13 **	−0.19 **	0.21 **
NEG	*−0.29* ** (−0.37 **)	−0.15 * (−0.09)	---	0.41 **	−0.33 **
AGRES	*−0.46* ** (−0.39 **)	−0.19 * (−0.20)	0.34 **(0.55 **)	---	−0.60 **
CONT	*0.47* * (0.46 **)	0.25 ** (0.14)	−0.31 **(−0.39 **)	−0.63 ** (−0.47 **)	---

Note: DISC = discipline; COMM = communication; NEG = negative affect; CONT = effortful control; AGRES = aggressive behavior. Below the diagonal, the italicized values are for boys, and the values in parentheses are for girls. * *p* < 0.05.; ** *p* < 0.01.

**Table 5:** Fit indices of the models.

Mod.	RMSEA	GFI	AGFI	ECVI	CAIC	χ^2^	d.f.	*p*	Δχ^2^	Δd.f.	*p*
1–3 Years Old											
1.1	0.33	0.73	0.50	0.97	230.50	216.25	8	<0.01	---	---	---
1.2	0.36	0.76	0.39	0.88	327.56	191.42	6	<0.01	---	---	---
1.3	0.36	0.92	0.38	0.38	149.28	53.98	2	<0.01	137.48	4	<0.01
1.1 Boys	0.28	0.79	0.60	0.79	135.46	93.92	8	<0.01			
1.2 Boys	0.28	0.80	0.49	0.78	141.58	88.17	6	<0.01			
1.3 Boys	0.29	0.93	0.50	0.37	102.03	24.88	2	<0.01	63.29	4	<0.01
1.1 Girls	0.42	0.68	0.40	1.62	198.08	127.82	8	<0.01			
1.2 Girls	0.45	0.71	0.27	1.46	188.12	111.36	6	<0.01			
1.3 Girls	0.38	0.90	0.26	0.55	106.41	29.52	2	<0.01	81.06	4	<0.01
3–6 Years Old											
2.1	0.30	0.77	0.58	0.78	256.93	210.24	8	< 0.01			
2.2	0.32	0.83	0.57	0.71	245.54	148.08	6	<0.01			
2.3	0.13	0.98	0.88	0.15	94.07	9.61	2	<0.01	236.47	4	<0.01
2.1 Boys	0.32	0.78	0.59	1.03	135.67	75.99	8	<0.01			
2.2 Boys	0.32	0.83	0.58	0.77	148.93	75.82	6	<0.01			
2.3 Boys	0.08	0.99	0.91	0.28	77.62	3.41	2	0.18	74.21	4	<0.01
2.1 Girls	0.35	0.76	0.54	1.13	222.14	139.45	8	<0.01			
2.2 Girls	0.29	0.83	0.57	0.66	217.83	78.37	6	<0.01			
2.3 Girls	0.17	0.97	0.80	0.22	91.85	11.55	2	<0.01	66.82	4	<0.01

Note: RMSEA: root mean square error of approximation; GFI: goodness of fit index; AGFI: adjusted goodness of fit Index; ECVI: expected cross-validation index; CAIC: Consistent Akaike’s Information Criterion.
